# Acute hepatitis E virus infection following ECMO-assisted cardiopulmonary resuscitation and blood transfusion: A case report and systematic review

**DOI:** 10.1097/MD.0000000000045382

**Published:** 2025-10-24

**Authors:** Junjun Wu, Jiabin Xiong, Kejie Hu, Yahong He, Zhen Li, Huaming Li, Yufang Wang, Rong Xu

**Affiliations:** a Department of Gastroenterology, Hangzhou Third People’s Hospital, Hangzhou, Zhejiang, China.

**Keywords:** abnormal liver function, acute hepatitis E, blood transfusion, immunocompromised, transmission route

## Abstract

**Rationale:**

In this province, ensuring hepatitis E virus (HEV)-free blood products is imperative. Monitoring HEV antibodies in symptomatic patients with abnormal liver function is key to preventing transfusion-induced hepatitis.

**Patient concerns:**

A 34-year-old female had a sudden cardiopulmonary arrest, treated with cardiopulmonary resuscitation, defibrillation, and extracorporeal membrane oxygenation (ECMO). She was discharged but developed liver dysfunction 15 days postsurgery, with positive HEV-IgM.

**Diagnoses:**

She was diagnosed with HEV infection. Considering her critical postresuscitation status during ECMO support, the blood transfusion received at that time is highly suspected as the potential route of HEV transmission.

**Interventions:**

The patient received immediate treatment with Baojians, a hepatoprotective agent, to reduce elevated liver enzymes. Serial monitoring of her liver function was conducted, including regular assessments of aminotransferases, bilirubin, and coagulation parameters. Additionally, a detailed review of her transfusion history was performed to identify potential sources of HEV exposure, such as previous blood product transfusions or organ transplants.

**Outcomes:**

The patient achieved complete recovery, with liver function parameters returning to normal ranges. This outcome not only validated the effectiveness of the implemented treatment regimen but also underscored the significant risk of HEV transmission via blood transfusion.

**Lessons:**

Blood transfusions in ECMO rescue enhance coagulation and manage anemia, but they also pose risks associated with transfusions. Among these, acute jaundice hepatitis E caused by blood transfusion requires our special attention. To reduce the incidence of hepatitis E from blood transfusions, it is advisable to incorporate hepatitis E antibody monitoring into blood product testing.

## 1. Introduction

Despite advances in cardiopulmonary resuscitation, sudden cardiac arrest remains a clinical emergency. Over 6 million people worldwide experience cardiac arrest each year, with a survival rate of <10%.^[1]^ Extracorporeal membrane oxygenation (ECMO), as a salvage treatment, provides temporary cardiopulmonary support for patients with refractory cardiac arrest to achieve hemodynamic stabilization.^[2]^ However, ECMO treatment often requires large blood transfusions due to pipeline-related coagulation dysfunction and blood dilution, exposing patients to the risk of transfusion-transmitted infections (TTIs).

Currently, blood screening strategies have effectively reduced the transmission of major pathogens, such as human immunodeficiency virus (HIV), hepatitis B virus (HBV), and hepatitis C virus (HCV). However, hepatitis E virus (HEV) a pathogen capable of being transmitted through blood transfusion, is an emerging concern, particularly in non-endemic areas. Genotypes 3 and 4 are mainly distributed in developed regions, and as zoonotic pathogens, pigs are the main host. HEV can cause acute or chronic hepatitis in immunosuppressed hosts. However, because blood donors often have asymptomatic infection, they have a high rate of missed diagnosis.^[3]^ Recent European^[4]^ studies have shown that the positive rate of HEV-RNA in blood donors is about 0.01 to 0.076%, although the risk of transmission cannot be ignored.

Although transmission of HEV through transfusion is gaining attention, reports of secondary HEV infection in patients with ECMO are extremely rare. This gap is concerning because these patients are at high risk of blood transfusions and may be immunocompromised after resuscitation. Herein, we report a case of acute hepatitis E in a patient with sudden cardiac arrest who was successfully treated with ECMO. Blood transfusion was identified as the source of the virus through traceability analysis. In addition, this study systematically reviewed the literature on HEV transmitted through blood products, providing a reference for ECMO management and blood safety policy. This case was approved by hospital ethics (2025KA120).

## 2. Case report

### 2.1. Chief complaint

Recurrent liver injury 15 days post-ECMO in a patient who sustained cardiac arrest for 1 hour.

### 2.2. History of present disease

The patient was a 34-year-old previously healthy female, who experienced cardiopulmonary arrest and was successfully resuscitated after 1 hour of cardiopulmonary pulmonary resuscitation. However, due to persistent cardiopulmonary shock, the patient was transferred to the intensive care unit at Hangzhou First People’s Hospital, where she received VA-ECMO support (January 28, 2022–February 2, 2022), endotracheal intubation mechanical ventilation (January 28, 2022–February 3, 2022), and other supportive treatment for diagnosis of “acute explosive myocarditis, acute respiratory failure, acute liver failure, acute kidney failure, hypoxic ischemic encephalopathy, anemia, lung infection, urinary tract infection.”

Supportive treatments included dobutamine for cardiac strength, norepinephrine as a vasoactive drug to maintain blood pressure, blood transfusion, and potassium supplementation to improve brain function, provide nutrition to the myocardium, and protect the liver. Other treatments included methylprednase injection (40 mg Q12H) to suppress inflammation storm, and piperacillin and tazobactam (4.5 g Q12H) were given as antibiotherapy for 8 days.

During follow-up, the patient’s body temperature was repeatedly measured and the a peak temperature was 38.7°C. In addition, the inflammatory index was increased. Bedside chest X-ray indicated exudative changes in the lower right lung, and a sputum culture on February 4th revealed the presence of *Klebsiella pneumoniae*. Based on drug sensitivity results, ertapenem 1.0 g Q8H combined with vancomycin injection 1 g Q12H as antibiotherapy for 6 days (February 4, 2022–February 9, 2022) was shown to be effective. After symptom relief, he was treated with piperacillin-tazobactam (4.5 g Q12H) combined with vancomycin (1 g Q12H) as antibiotherapy (February 4, 2022–February 9, 2022). The patient remained stable and was transferred to our hospital on day 12 for further treatment.

### 2.3. Physical examination after admission

On admission, the patient’s body temperature was 37.6°C, pulse was 98 times/min, respiratory rate was 20 breaths/min, and blood pressure was 114/56 mm Hg. The patient was alert, oriented, and had a normal mental status. Thick breathing sounds were perceived in both lungs, with no obvious dry and wet rales heard. Heart rhythm and sounds were normal and no noise or murmurs were heard in each valve area. There was abdominal tenderness but no abdominal varicose vein, gastrointestinal type, or peristaltic wave. The liver and spleen were not palpable. Murphy sign was negative, and there was no shifting dullness. The frequency of bowel sounds was 3 times/min, and there was no tenderness and rebound pain. There was no edema in both lower limbs, and bilateral dorsal foot arteries were accessible.

### 2.4. Postoperative complications

On day 22 after ECMO withdrawal, the patient gradually developed elevated aminotransferase (alanine aminotransferase [ALT] 938 U/L, aspartate aminotransferase [AST] 540 U/L), jaundice, and coagulation dysfunction (prothrombin time [PT] 14.5 seconds). Electrocardiogram (ECG) revealed idiopathic ventricular fibrillation, prompting subcutaneous implantable cardioverter-defibrillator implantation. Drug-induced liver injury, ischemic hepatitis, and other hepatotropic virus infections (HAV-IgM, HBV-DNA, HCV-RNA negative) were ruled out.

### 2.5. Laboratory test

On day 1 (Hangzhou First People’s Hospital), the biochemistry results were as follows: ALT 328 U/L, AST 712 U/L, procalcitonin 4.32 ng/mL, troponin I 0.064 µg/L. Lymphocyte subgroup typing revealed CD4 at 247.88 × 10^6^/L and CD3 + at 539.78 × 10^6^/L. By day 11, the patient’s biochemistry results were as follows: ALT 273 U/L, AST 228 U/L, total bilirubin 19.2 μmol/L, urea 7.48 mmol/L, creatinine 128 μmol/L, procalcitonin 4.32 ng/mL, and troponin I8.60 μg/L. Immunological tests revealed lgG at 5.75 g/L, lgA at 1.68 g/L, complement C3 at 0.30 g/L, complement C4 at 0.04 g/L. Blood gas analysis showed PH 7.42, BE-5.5, PaO_2_ 463.3 mm Hg, PaCO_2_ 27.8 mm Hg, bicarbonate 17.8 mmol/L, and potassium 1.9 mmol/L. Sputum culture identified *K pneumoniae*, while urine culture identified vancomycin-sensitive *Enterococcus faecium*: (10^4^–10^5^ CFU/mL).

On day 12 (Hangzhou Third People’s Hospital), emergency biochemical findings were as follows: ALT 74 U/L↑, amylase 485 U/L↑, AST 39 U/L↑, calcium 1.97 mmol/L↓, creatine kinase 245 U/L↑, glucose 7.98 mmol/L↑. Emergency blood routine and C-reactive protein (CRP) revealed CRP at 6.5 mg/L (0–10 mg/L), white blood cells at 7.3 × 10^9^/L, hemoglobin at 71 g/L↓ (115–150 g/L), platelets at 162 × 10^9^/L (100–300 × 10^9^/L), and neutrophil percentage at 79.5%↑.

On day 15, routine blood tests revealed red blood cells at 2.11 × 10^12^/L↓ and hemoglobin at 64 g/L↓. Lymphocyte subgroup typing revealed CD8 + at 513 × 10^6^/L, CD4 + at 738 × 10^6^/L, B lymphocytes at 10.6%, NK cells (CD56+) at 59 × 10^6^/L↓, T lymphocytes percentage at 85.7%↑, and NK cells percentage at 3.9%↓. Tests for hepatitis B and hepatitis C antibodies, syphilis and HIV antibodies, and autoimmune liver disease antibodies were all negative.

On day 20, liver function tests revealed albumin at 33.5 g/L↓, alkaline phosphatase at 109 U/L↑, ALT at 155 U/L↑, amylase at 221 U/L↑, AST at 111 U/L↑, creatinine at 49 μmol/L, glutamyl transphthalase at 46 U/L↑, potassium at 3.72 mmol/L, and lactate dehydrogenase at 367 U/L↑.

On day 26, coagulation tests showed PT at 14.1 seconds and D-dimer (quantitative) 1.48 mg/L↑. Biochemical tests revealed albumin at 34.8 g/L↓, alkaline phosphatase at 159 U/L↑, ALT at 763 U/L↑, amylase at 139 U/L↑, AST at 539 U/L↑, creatine kinase at 25 U/L↓, CK isoenzyme at 12 U/L, creatinine at 52 μmol/L, direct bilirubin at 8.6 μmol/L↑, and glutamyl transferase at 116 U/L↑, immunoglobulin M at 3.03 g/L↑, potassium 3.77 mmol/L, lactate dehydrogenase at 615 U/L↑. Additional coagulation tests revealed activated partial thromboplastin time at 34.6 seconds, D-dimer (quantitative) at 1.48 mg/L↑, INR at 1.19, and PT at 13.3 seconds.

**Figure 1.**
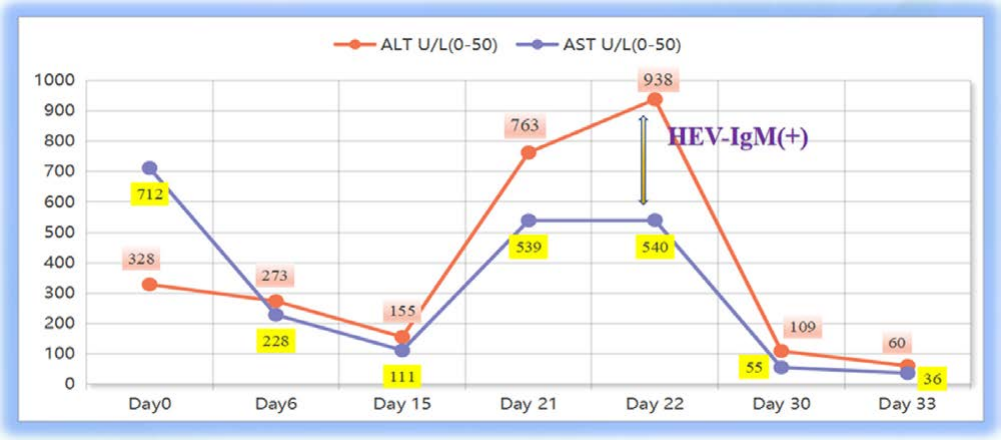
Trends in levels of alanine aminotransferase (ALT) and aspartate aminotransferase (AST). HEV = hepatitis E virus.

**Figure 2.**
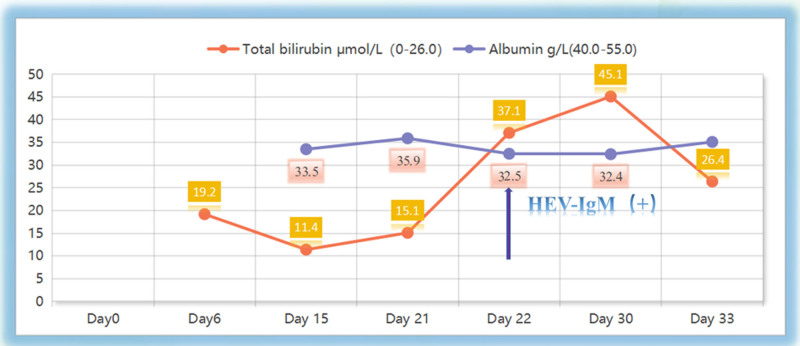
Trends in levels of total bilirubin, albumin. HEV = hepatitis E virus.

**Figure 3.**
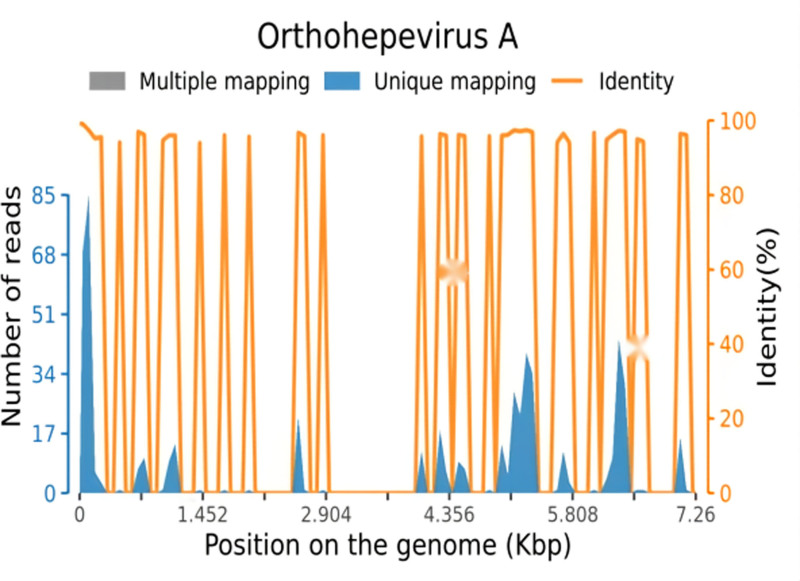
Sequence map of HEV cultured using next-generation sequencing (NGS). NGS identified HEV genotype 4 (read depth: 98.7%), confirming acute infection. HEV = hepatitis E virus.

On day 27, liver function tests revealed albumin at 35.9 g/L↓, alkaline phosphatase at 187 U/L↑, ALT at 938 U/L↑, AST at 540 U/L↑, direct bilirubin at 29.0 μmol/L↑, glutamyl transphthalase at 154 U/L↑, potassium at 3.92 mmol/L, total bilirubin at 37.1 μmol/L↑. Coagulation tests showed activated partial thromboplastin time at 38.5 seconds↑, D-dimer (quantitative) at 1.53 mg/L↑, INR at 1.26↑, and PT at 14.1 seconds↑ (Figs. 1 and 2, Tables 1 and 2). Serum HEV-IgM was positive in the acute phase (ELISA, Wantai Bio), and serum next-generation sequencing detected HEV (Fig. 3). HEV-IgM antibody positive lasted for 3 months during the recovery period, which was consistent with the serological dynamics of acute HEV infection.

**Table 1:** Specific values of liver function, bilirubin, albumin, and prothrombin time during the onset of the disease.

Laboratory parameters	Post-ECMO	ALT U/L (0–50)	AST U/L (0–50)	Total bilirubin μmol/L (0–26.0)	Direct bilirubin μmol/L (0–8.0)	Albumin g/L (40.0–55.0)	Prothrombin time (s) (9.0–14.0)	HEV-IgM
Day 1	Day 0	328	712					Negative
Day 11	Day 6	273	228	19.2				
Day 12	Day 7	74	39				11.1	
Day 13	Day 8	78	31	11.8	4.7	32.1		
Day 14	Day 9	62	23	12.8	5.3	30.7		
Day 20	Day 15	155	111	11.4	4.5	33.5		
Day 21	Day 16	193	108	9.3	3.7	34.4		
Day 23	Day 18	183	87	9.8	3.7	36.2		
Day 25	Day 20	536	394	14.9	7.1	34.8		
Day 26	Day 21	763	539	15.1	8.6	35.9	13.3	
Day 27	Day 22	938	540	37.1	29	32.5	14.1	Positive
Day 28	Day 23	748	323	50.6	42.7	33	14.5	
Day 29	Day 24	553	240	61.2	52.6	33.2	13.7	
Day 30	Day 25	275	201	69.3	62.5	31.3	12.7	
Day 33	Day 28	171	104	63.6	58.7	30.9		
Day 35	Day 30	109	55	45.1	42	32.4		
Day 38	Day 33	60	36	26.4	23.4	35.1	10.5	
Day 49	Day 44	34	27	14.6	10.5	40.0	11	

ALB = albumin, ALT = alanine aminotransferase, AST = aspartate aminotransferase, PT = prothrombin time, TB = total bilirubin.

**Table 2:** Temporal trends in key laboratory parameters.

Post-ECMO	ALT U/L (0–50)	AST U/L (0–50)	Total bilirubin μmol/L (0–26.0)	Direct bilirubin μmol/L (0–8.0)	Albumin g/L (40.0–55.0)	Prothrombin time (s) (9.0–14.0)	HEV-IgM
Day 0	328	712					Negative
Day 6	273	228	19.2				
Day 15	155	111	11.4	4.5	33.5		
Day 21	763	539	15.1	8.6	35.9	13.3	
Day 22	938	540	37.1	29	32.5	14.1	Positive
Day 30	109	55	45.1	42	32.4		
Day 33	60	36	26.4	23.4	35.1	10.5	
Day 44	34	27	14.6	10.5	40	11	

ALT = alanine aminotransferase, AST = aspartate aminotransferase, HEV = hepatitis E virus.

### 2.6. Imaging examination

ECG revealed sinus rhythm, ST-segment elevation (I, II, III, aVF, and V2-V6 elevation [0.03–0.05 mV]), and T-wave changes. A bedside chest X-ray showed slight inflammatory exudation of the right middle lung. Pituitary magnetic resonance with enhancement revealed no significant abnormalities. B-mode ultrasonography was performed, with bedside cardiac ultrasonography showing no obvious abnormality. No obvious abnormality was found in lower limb arteries and deep venous blood flow in both lower limbs was normal.

**Figure 4.**
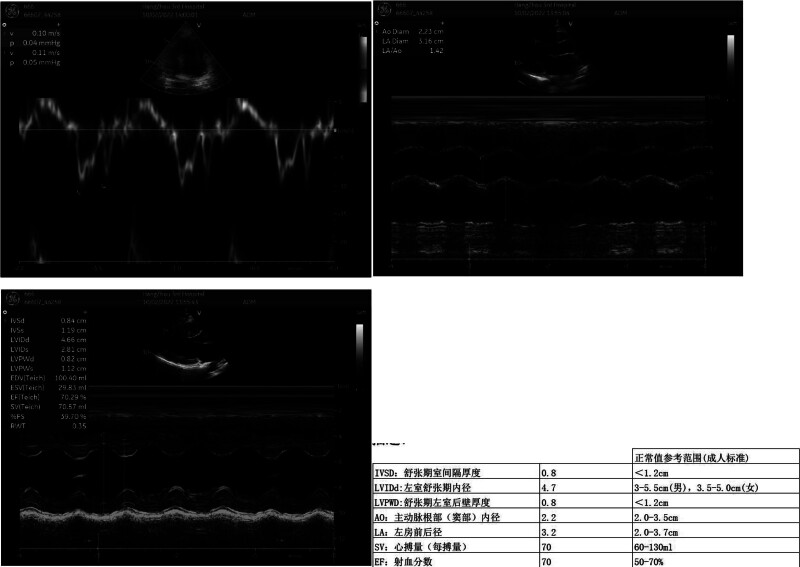
Echocardiography report during recovery.

On day 13 a cardiac ultrasound at Hangzhou Third People’s Hospital indicated mild regurgitation of mitral, tricuspid, and pulmonary valves (EF 70%) (Fig. 4), and no abnormality was found in normal computed tomography scan of the upper abdomen.

**Figure 5.**
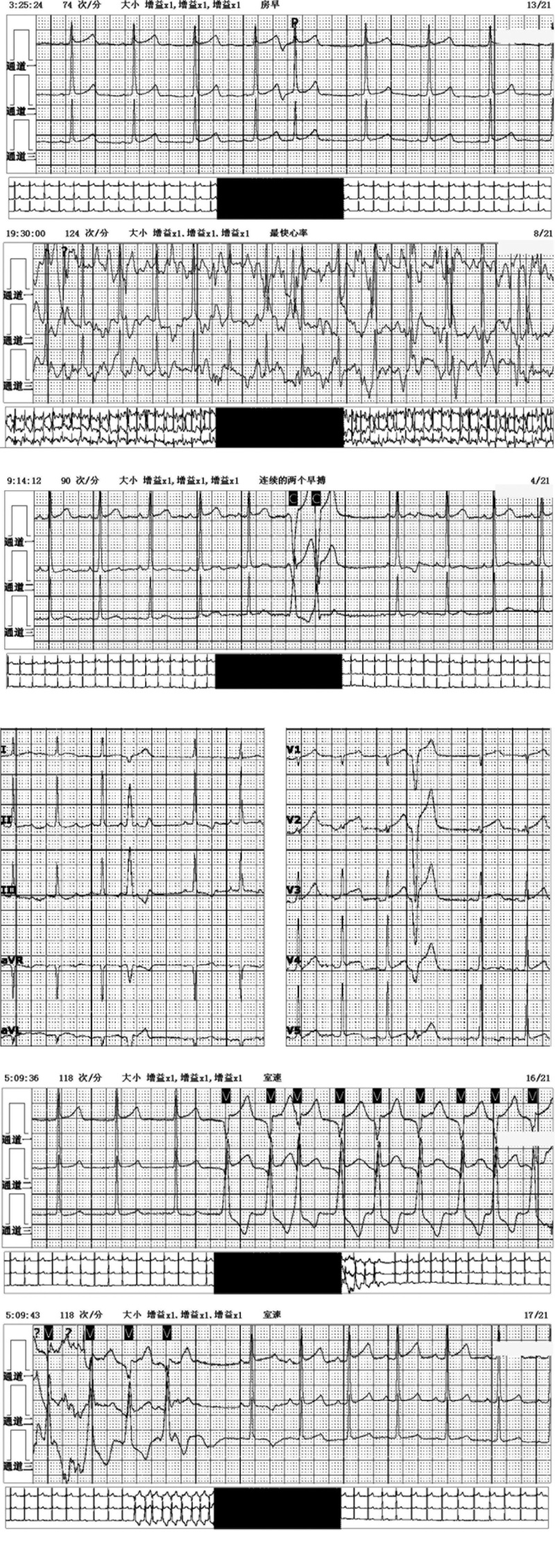
The 24-hour Holter electrocardiogram indicated frequent ventricular tachycardia, ventricular premature beat, and atrial premature beat.

On day 52, 24-hour Holter ECG showed sinus rhythm, occasional premature ventricular beats with ventricular bradycardia, atrial premature beat, intermittent T-wave changes, and intermittent STch1 elevation (Fig. 5).

### 2.7. Blood transfusion history

During ECMO treatment, 8.5 U of red cell suspension and 1810 mL of plasma products were transfused. The blood products passed routine screening (negative for HIV, HBV, HCV, and syphilis). However, HEV-RNA testing was not performed, as itis not routinely included in blood bank screening in this region. Epidemiological investigation in China: patients with no history of travel, no consumption of undercooked meat or shellfish, and no known contact with HEV.

### 2.8. Treatment and outcome

**Figure 6.**
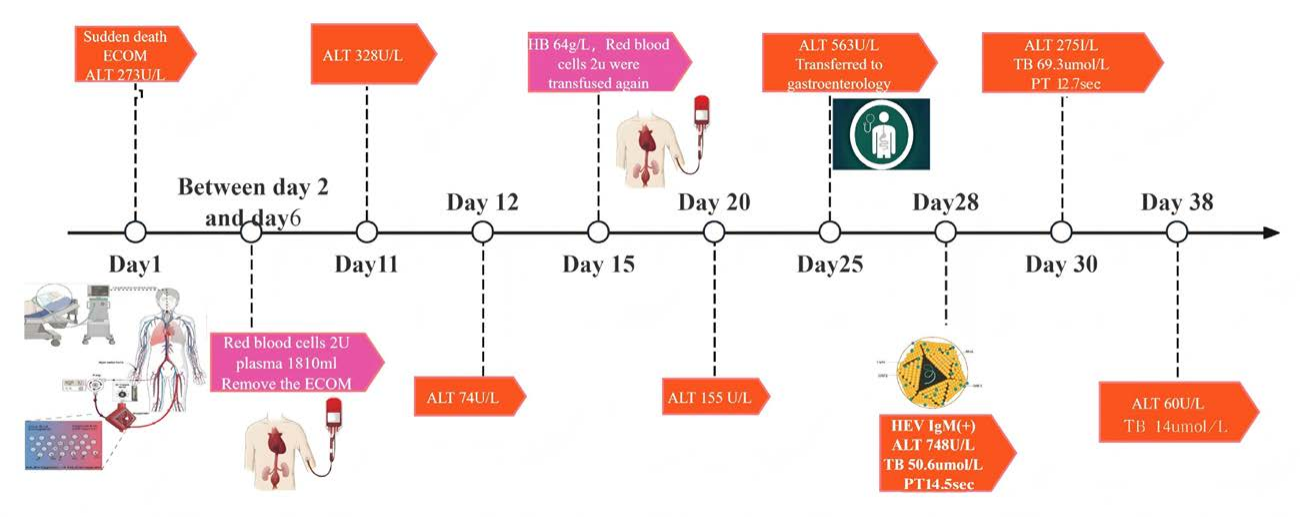
Complete diagnosis and treatment process for the patient. ALT = alanine aminotransferase, ECMO = extracorporeal membrane oxygenation, HEV = hepatitis E virus, PT = prothrombin time.

The patient was treated with 150 mg qd + of polyene phosphatidylcholine and 465 mg qd + of adenosine butyldisulfonic acid 1 g, with close monitoring. No ribavirin was used. Liver function gradually recovered 38 days after onset (ALT decreased to 60 U/L), and there were no signs of chronicity after 3 months of follow-up (Fig. 6). The disease was stable after implantable cardioverter-defibrillator implantation, and no malignant arrhythmia occurred.

### 2.9. Ethical approval

This study was approved by the Ethics Committee of Hangzhou Third People’s Hospital (Approval No.: 2025KA120). Written informed consent was obtained from the patient for the publication of this case report and accompanying images.

## 3. Discussion

This case highlights 2 key issues in modern critical care. First is the risk of HEV transmission through blood products in patients requiring considerable blood transfusions, such as those receiving ECMO. While HEV infection is primarily transmitted through food and water, transfusion-related transmission has been reported in developing and developed countries. In immunocompromised patients, HEV infection can lead to chronic hepatitis E, progressing to fibrosis and cirrhosis.^[5,6]^ Additionally, the risk of transfusion-transmission HEV depends on factors such as the frequency of viremia in blood donors, viral load, and plasma volume in the final transfusion blood component.^[7]^ In the UK, to protect high-risk patients, blood components are provided only from donors screened for HEV viremia. However, this approach does not prevent HEV exposure through diet, raising questions about the relative risk of transmission via transfusion versus dietary sources.^[8]^ Given these uncertainties, policymakers should consider implementing frameworks in transfusion medicine to guide the appropriate preventive measures and improve blood safety.^[9]^ These measures can help mitigate the risk of transfusion-associated HEV transmission, especially in critically ill patients who require extensive transfusion support.

Second, in immunocompromised patients, HEV infection can persist and manifest as a chronic disease, which is related to the virus’ ability to persist in the presence of interferon. This persistent infection may be due to the virus evading the cell’s antiviral defense mechanisms.^[10]^ In addition, HEV infection in immunocompromised patients can lead to serious complications, such as neurological and kidney diseases.^[11]^ In terms of blood safety, although HEV infection is usually self-limited, it can lead to serious health consequences in immunocompromised individuals. Therefore, selective screening for high-risk groups is necessary, especially in areas where HEV is prevalent.^[12,13]^ Moreover, the diagnosis of HEV infection should include the application of molecular techniques to enable more accurate detection in immunosuppressed patients.^[14]^

### 3.1. ECMO and HEV infection risk

After ECMO surgery, patients may face multiple complications, with acute viral hepatitis E being an important concern. Studies have shown that patients who have undergone ECMO surgery are at significantly increased risk of HEV infection due to immunosuppression and potential impairment of liver function. In a case analysis, the postoperative patient developed symptoms of acute hepatitis after blood transfusion, with further testing confirming HEV infection. This case usually presents with jaundice, liver dysfunction, and viral RNA positivity, and in immunosuppressed patients, HEV infection may rapidly progress to chronic hepatitis, leading to more severe liver damage.^[15]^ In addition, the study found that some patients infected with HEV after surgery were able to clear the virus after receiving antiviral therapy, but others were at a higher risk of death due to liver failure.^[16]^ Therefore, monitoring and early diagnosis of HEV infection in patients post-ECMO surgery should be strengthened to enable timely intervention. In our case, the patient developed abnormal liver function 15 days after ECMO and serum next-generation sequencing testing was positive for HEV virus, ruling out other infectious causes.

### 3.2. Diagnostic challenges and immunosuppressive mechanisms

The 22-day interval between transfusion and onset of symptoms reflects the incubation period of HEV (15–60 days).^[17]^ Notably, HEV-IgM was consistently negative until the onset of jaundice, possibly due to ECMO-induced immunosuppression. Similar seroconversion delays have been reported in transplant recipients, and RNA testing is considered the gold standard for diagnosis.

A retrospective analysis of 23 cases of transfusion-associated hepatitis E (TTIs-HEV) in France between 2006 and 2016 showed that many of the patients were organ transplant recipients or patients with malignant blood diseases, and most of the cases were associated with transfused plasma products, especially solvent-treated plasma.^[16]^ The HEV-RNA load in these cases was significantly higher than in uninfected persons, suggesting that transmission of HEV is closely related to the viral load of the transfused product.^[18]^

In some cases, the infection may remain asymptomatic until weeks after the transfusion, further complicating clinical diagnosis. Therefore, it is necessary to implement strict blood screening and monitoring measures for high-risk patients, especially immunosuppressed patients, to reduce the risk of transmission of TTIs-HEV and improve patient outcomes.^[19]^ This case advocates active HEV-IgM surveillance in transfusion recipients with unexplained hepatitis, even if serological results are negative.

### 3.3. Immunosuppressed status and susceptibility to HEV

Patients with ECMO are often immunosuppressed due to critical primary disease, surgical trauma, and the use of immunosuppressive drugs, increasing the risk of HEV infection or progression to chronic infection. Recent studies suggest that impaired immune function is the key risk factor for chronic and severe HEV infection, without several mechanisms contributing to this susceptibility.

#### 3.3.1. Characteristics of HEV infection in immunosuppressed patients

In people with normal immune function, HEV infection usually presents as self-limited acute hepatitis. However, viral clearance is reduced in immunosuppressed patients, such as organ transplants, patients with hematological disorders,^[15]^ and long-term immunosuppressant users, significantly increasing the risk of chronic disease. Chronic HEV infection is defined as viremia lasting more than 3 months. The risk of progression to liver failure and death in patients with chronic liver disease are 35.88% and 14.33%, respectively.^[20]^ We previously identified an elderly patient who develop liver failure after HEV infection and eventually died from a secondary fungal infection.^[21]^

#### 3.3.2. Immunosuppressive mechanisms and persistent HEV infection

HEV clearance depends on host T-cell-mediated immune responses, especially CD4 + and CD8 + T-cells. Studies have shown that HEV infection significantly inhibits the CD8 + T cell activity, leading to T-cell dysfunction.^[22]^ Additionally, HEV infection reduces the host’s ability to produce antigen-specific antibodies, which may be due to structural changes in the viral antigen or impaired B-cell function.^[23]^ Immunosuppressants, such as calcineurin inhibitors and glucocorticoids, can further inhibit T-cell function, weaken virus-specific immune responses, and lead to uncontrolled viral replication.^[24,25]^ Moreover, in immunosuppressed individuals, suppression of innate immunity, such as inhibition of interferon signaling pathways, may contribute to persistent HEV infection.^[26,27]^ Furthermore, estrogen-ERα66 signaling and STAT3 co-regulate SOCS3 expression, which may regulate HEV-induced interferon response in rabbits.^[28]^

#### 3.3.3. Inflammatory-nutritional biomarkers and prognostic value

The interplay between immune suppression and nutritional status in transfusion-related HEV infection can be further supported by recent studies on composite biomarkers. The Onodera prognostic nutritional index, which combines serum albumin and lymphocyte count, has been shown to predict postoperative complications and long-term survival in colorectal cancer patients when integrated with inflammatory markers.^[29]^ Similarly, the pan-immuno-inflammatory value combined with albumin-to-globulin ratio was identified as a reliable indicator for assessing systemic inflammation and nutritional status, with low pan-immuno-inflammatory value–albumin-to-globulin ratio correlating with poor outcomes.^[30,31]^ For critically ill patients like ours (who presented with hypoalbuminemia [33.5 g/L↓], reduced lymphocyte count [CD4 + 247.88 × 10^6^/L initially], and elevated CRP, these indices could serve as surrogate markers for predicting HEV-related liver injury severity.

Additionally, the platelet–albumin ratio and cancer inflammation prognostic index have been validated in multiple cohorts as predictors of infectious complications and mortality.^[32]^ Notably, bibliometric analyses of immune-related complications in cancer immunotherapy highlight that systemic inflammatory responses (e.g., elevated CRP, reduced complement levels) are independent risk factors for poor prognosis^[33]^: a finding consistent with our patient’s clinical course (persistent inflammation, hypocomplementemia). These biomarkers collectively emphasize that monitoring immune-nutritional status is critical for early identification of high-risk transfusion-related HEV patients and guiding intervention.

### 3.4. Clinical presentation and diagnostic challenges

HEV infection in immunosuppressed patients is often characterized by mild aminotransferase elevation or lack of typical hepatitis symptoms, which is easily masked by the primary disease, leading to delayed diagnosis.^[34]^ Therefore, timely detection of HEV-RNA (serum or fecal) and anti-HEV-IgM/IgG is recommended for immunosuppressed patients with unexplained liver dysfunction or thrombocytopenia.

### 3.5. Clinical significance of ECMO management

Our patients recovered without antiviral therapy, which may be due to differences in viral load, host immunity, or comorbidities. Importantly, ribavirin, a 1st-line treatment for chronic HEV, is considered contraindicated in these patients due to potential drug interactions with post-resuscitation drugs such as amiodarone. This dilemma underscores the importance of hepatitis E prevention rather than reactive treatment in severely ill patients.

### 3.6. Blood safety policy recommendations

TTIs-HEV has attracted increasing clinical attention in recent years, especially in immunosuppressed patient.^[3]^ Multiple studies have shown that blood transfusion is an important route of transmission of TTIs-HEV, especially in patients who have received multiple transfusions.^[16,35]^ The detection of transfusion-associated infectious viruses should be strengthened, and HEV testing of blood donators^[36]^ has been carried out in several countries in Europe, including the Netherlands, where HEV testing of blood donors prevents approximately 4.52 cases of transfusion-related HEV infection per year, at a cost of approximately €310,000 per prevented case.^[37]^ Despite the relatively high cost of HEV screening, it remains comparable to other blood screening measures. Despite the low HEV-RNA detection rate in large samples, Australian researchers recommend enhanced HEV screening for high-risk populations, such as immunosuppressed individuals.^[38,39]^ This case further demonstrates that strengthening screening of the virus in blood donors can effectively reduce the occurrence of related infectious diseases.

The diagnostic and therapeutic process of this case has further deepened our understanding of transfusion-related infectious diseases. In clinical practice, for patients with unexplained liver damage who have a history of blood transfusion, when conditions permit, we should actively trace the results of HEV detection in blood donation samples and blood donors to provide a key basis for etiological diagnosis.

The confirmation of transfusion-transmitted HEV requires 2 core conditions: it is clear that the blood donor is in the HEV viremic phase (i.e., positive for HEV-RNA detection); the HEV genotypes infected by the blood donor and the recipient are highly matched. However, in this case, since HEV has not been included in the routine screening items for blood donors in the local area, it is impossible to confirm whether the blood donors of the transfused blood products are HEV carriers. It is worth noting that the HEV viremic phase often has no obvious clinical symptoms, and the occult infection of blood donors is easily missed. The currently routinely carried out screening for pathogens transmitted through blood (HIV, HBV, HCV, syphilis) does not include HEV, which forms a potential screening gap in blood transfusion safety.

**Figure 7.**
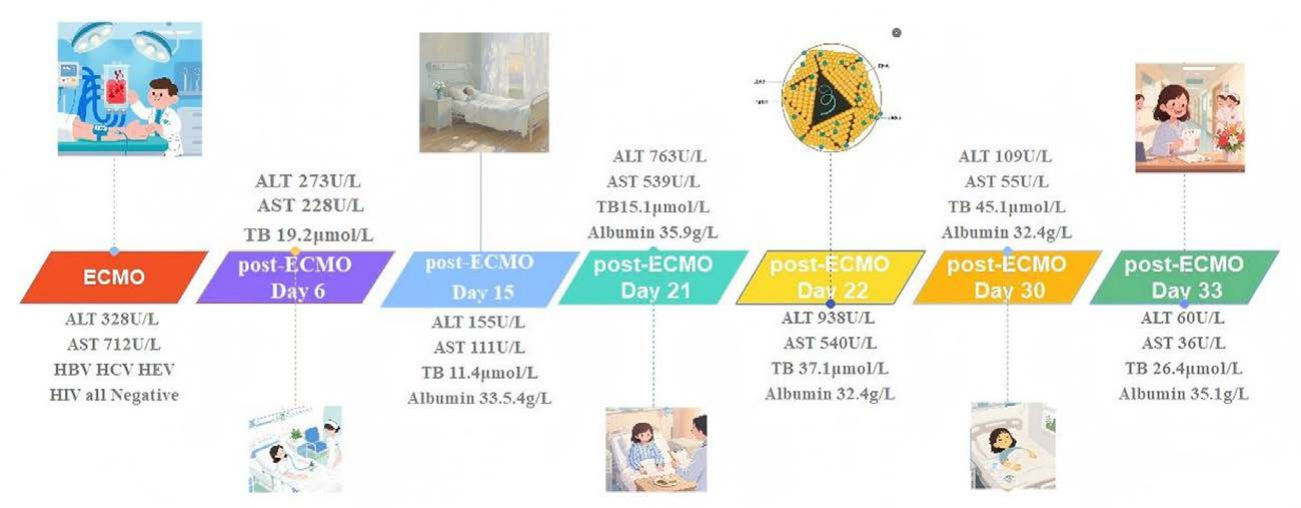
Illustrates the temporal progression of HEV infection following ECMO support, delineating the post-procedural seroconversion window and hepatic dysfunction milestones. ALT = alanine aminotransferase, AST = aspartate aminotransferase, ECMO = extracorporeal membrane oxygenation, HBV = hepatitis B virus, HCV = hepatitis C virus, HEV = hepatitis E virus, HIV = human immunodeficiency virus, PT = prothrombin time.

In addition, the diagnosis and treatment of the patient in this case involves 2 medical institutions, which is restricted by multiple factors. On the one hand, China’s strict privacy protection policies for blood donors restrict retrospective investigations; on the other hand, according to routine regulations, the storage period of blood samples is 30 days. The patient developed abnormal liver function 22 days after blood transfusion (Fig. 7), and when HEV infection was confirmed, it had exceeded the sample storage period, making it impossible to conduct retrospective testing on the retained samples of the corresponding blood donors. At the same time, there is a time mismatch between the 15 to 60-day incubation period of HEV and the existing sample storage period, which further increases the difficulty of retrospective verification.

Although this study excluded HEV infection caused by diet (such as raw meat/shellfish), travel and close contact through detailed epidemiological investigation, indirectly supporting the possibility of transfusion-transmission, it still cannot completely rule out the risk of occult exposure (such as polluted water sources, undetected indirect contact, etc). In addition, the patient’s immunocompromised state may prolong the virus incubation period or change the clinical phenotype, and these factors all increase the complexity of judging the infection route.

Despite the above limitations, this case still has important clinical implications: in the management of critically ill patients requiring massive blood transfusion such as those on ECMO, HEV should be listed as a key pathogen for screening transfusion-related infections. Based on this, we suggest: promote routine HEV-RNA screening for blood donors in HEV-endemic areas and high-risk groups, especially for immunocompromised patients who are to receive blood transfusion, and strengthen HEV detection for both donors and recipients; for viruses with long incubation periods such as HEV, appropriately extend the storage period of blood samples to 90 days to reserve a time window for retrospective testing; for patients with unexplained abnormal liver function after blood transfusion, timely carry out HEV marker detection to avoid missed diagnosis and misdiagnosis.

## 4. Conclusion

In conclusion, the inference of “transfusion-transmitted hepatitis E” in this case still relies on clinical logical chains and indirect evidence. In the future, it is necessary to improve the HEV detection system for blood donors and establish a traceability mechanism that balances privacy protection and public health safety to fill the gap in the evidence chain and provide a more solid scientific support for the optimization of blood transfusion safety policies.

## Acknowledgments

We sincerely thank the patient for her support and consent to publish this case. We also appreciate Doubao for providing image materials and polishing assistance (confirmation of consent to be acknowledged has been obtained).

## Author contributions

**Conceptualization:** Junjun Wu, Yufang Wang.

**Data curation:** Jiabin Xiong, Kejie Hu, Rong Xu.

**Formal analysis:** Yahong He.

**Investigation:** Junjun Wu, Jiabin Xiong, Yahong He, Rong Xu.

**Methodology:** Junjun Wu, Yahong He, Huaming Li, Yufang Wang.

**Project administration:** Junjun Wu, Huaming Li.

**Resources:** Zhen Li.

**Supervision:** Zhen Li.

**Validation:** Zhen Li.

**Visualization:** Huaming Li.

**Writing – original draft:** Junjun Wu.

**Writing – review & editing:** Yufang Wang.
